# Supplementary material for: In Silico Drug Repurposing Approach: Investigation of Mycobacterium tuberculosis FadD32 Targeted by FDA-Approved Drugs
Source: Molecules. 2022 Jan 20;27(3):668. doi: 10.3390/molecules27030668 (PMC8840176; doi:10.3390/molecules27030668)
Supplement: Supplementary file 1 [file molecules-27-00668-s001.zip › molecules-1480390-supplementary.pdf]

Supplementary Material

Table S1: Bind sites and the amino acids residues.

**Binding sites**	**Amino acids**
ATP-binding site	Thr187, Arg192, Glu347, Arg483, Asp469, Ala346, Ile480, Tyr343, Ser342, Pro316, Ser314.
Meromycolate-binding site	Val280, Met233, Ser342, Leu350, val211, Leu240, His246, Ile278, Thr237, Leu215.
Phosphopantetheine-binding site	Phe284*

* Phe284 is located at the entrance of the putative pantetheine-binding tunnel of FadD32.

Table S2: H bonding data of the investigated drugs.

**FDA-approved drugs**	**Acceptor**	**DonorH**	**Donor**	**Fra (ps)**	**Frac (%)**	**Dist (Å)**	**Angle (°)**
Accolate	PRO_194@O	ZIN_631@H	ZIN_631@N1	10357	0.052	2.88	156.91
Sorafenib	ZIN_631@O15	THR_191@HG1	THR_191@OG1	10811	0.054	2.74	159.04
Mefloquine	ZIN_631@F3	ARG_192@HH11	ARG_1942@NH1	1	0.000	2.87	146.31
	GLU_437@0E1	ZIN_631@H10	ZIN_631@N1	41586	0.208	2.87	157.74
Loperamide	ZIN_631@O1	ARG_192@HH22	ARG_192@NH2	3936	0.019	2.87	154.57
	ZIN_631@O1	ARG_192@HH12	ARG_192@NH1	2245	0.011	2.87	155.88
	ZIN_631@O	LYS_434@H	LYS_434@N	16	0.000	2.93	154.34

Fra – Frame; Frac – Fraction; Dist - Distance.

“Three replicas to verify the findings”

1. Binding free energy calculations

Table S3: Binding free energy (kcal/mol) and the components of binding free energy of the respective molecules.

FDA-approved drugs	ΔG_{bind} (kcal/mol)	ΔE_{vdw} (kcal/mol)	ΔE_{ele} (kcal/mol)	ΔG_{gas} (kcal/mol)	ΔG_{sol} (kcal/mol)
**Accolate**	-45.13±6.64	-64.54±4.08	-28.89±9.70	-93.44±12.41	48.31±6.95
	-54.15±6.06	-70.40±3.68	-37.67±7.86	-108.08±8.79	53.39±4.79
	-48.96±3.86	-57.29±2.85	-51.00±8.22	-108.29±7.79	59.33±4.77
**Average**	-49.29±5.52	-63.66±3.53	-39.19±8.59	-103.27±9.66	53,67±5.50
**Sorafenib**	-32.73±3.87	-51.64±2.92	-27.74±8.37	-73.37±8.36	46.65±7.83
	-48.98±3.38	-58.71±3.19	-43.05±6.21	-101.76±6.56	52.77±4.05
	-42.60±6.32	-63.78±4.82	-29.49±8.74	-93.47±11.29	50.87±7.34
**Average**	-41.43±4.52	-58.04±3.64	-33.42±7.77	-89.53±8.73	50.09±6.41
**Mefloquine**	-26.84±2.63	-34.55±2.74	-23.59±6.18	-58.13±6.15	31.29±4.97
	-21.13±2.93	-30.87±3.37	-12.81±6.18	-43.68±7.97	22.54±6.43
	-39.61±5.77	-48.88±4.41	-37.06±8.54	-85.95±11.30	46.33±7.26
**Average**	-29.19±3.77	-38.10±3.51	-24.48±6.96	-62.58±8.47	33.38±6.22
**Loperamide**	-21.52±7.40	-33.45±7.95	-11.37±7.48	-59.84±10.21	28.39±6.01
	-36.07±3.19	-47.98±2.81	-29.29±7.48	-77.90±4.59	41.83±3.21
	-20.79±4.31	-33.25±4.10	-18.11±8.05	-51.36±9.38	30.57±7.29
**Average**	-26.37±4.96	-38.22±4.95	-19.59±7.67	-63.03±8.06	33.59±5.50

2. Systems stability

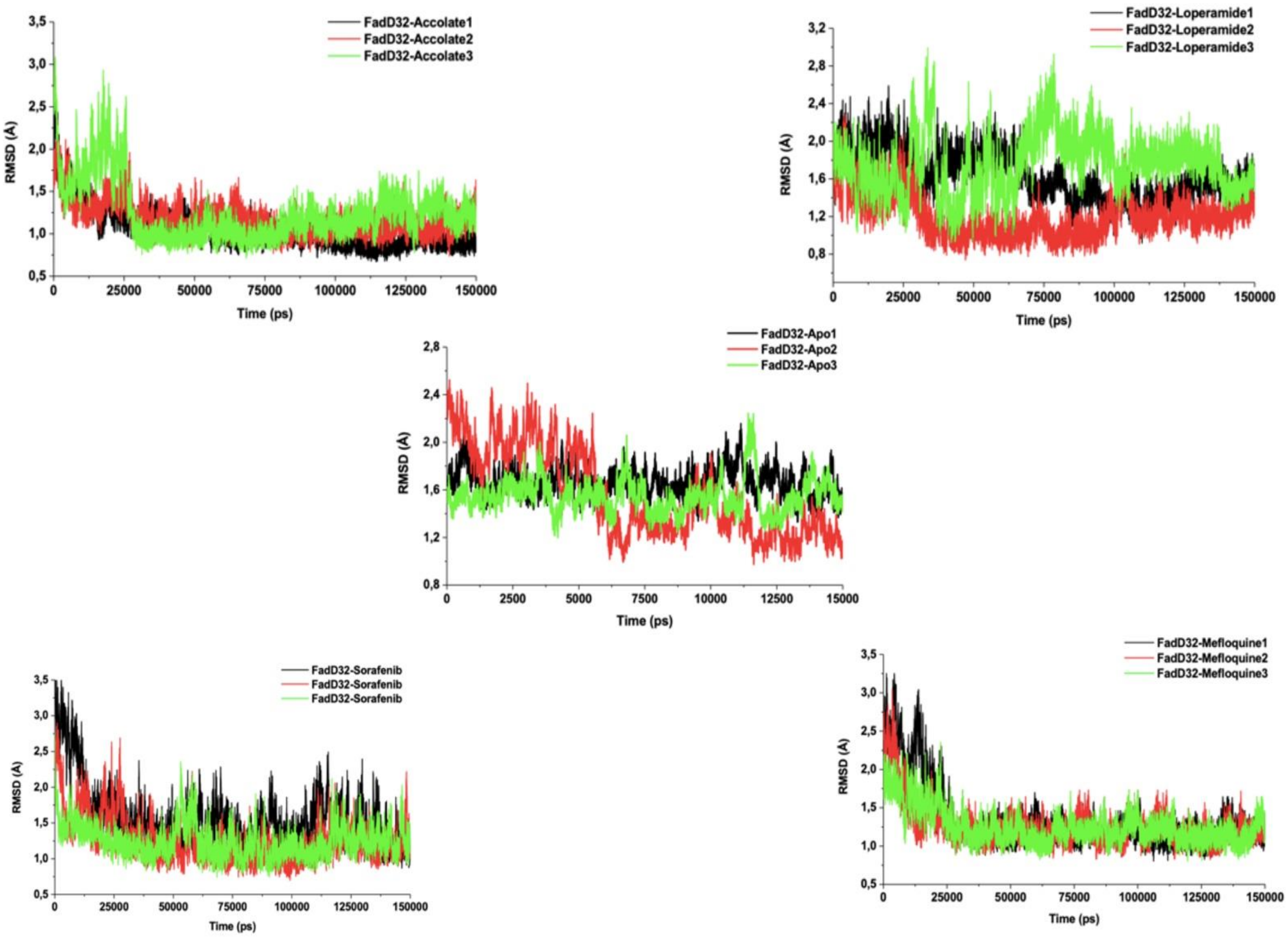

Figure S1: RMSD of FadD32-Apo, FadD32-Accolate, FadD32-Sorafenib, FadD32-Mefloquine and FadD32-Loperamide

Table S4: RMSD (Å) values and average.

	#1	#2	#3	Average
**APO**	1.20	1.36	1.38	1.31
**Accolate**	1.05	1.18	1.24	1.16
**Sorafenib**	1.56	1.26	1.25	1.36
**Mefloquine**	1.35	1.24	1.24	1.28
**Loperamide**	1.63	1.21	1.77	1.55
